# La enseñanza de la Ley Nacional de Trasplante como camino ético para la construcción del valor de la atención en salud en Colombia

**DOI:** 10.7705/biomedica.7388

**Published:** 2024-08-29

**Authors:** Juan Bernardo Hoyos

**Affiliations:** 1 Programa de Doctorado en Bioética, Facultad de Educación y Humanidades, Universidad Militar Nueva Granada, Cajicá, Colombia Universidad Militar Nueva Granada Universidad Militar Nueva Granada Cajicá Cajicá

**Keywords:** trasplante de órganos, obtención de tejidos y órganos, bioética, educación, consentimiento informado, voluntad en vida., Organ transplantation, tissue and organ procurement, bioethics, education, informed consent, living wills

## Abstract

La Ley Nacional de Trasplantes en Colombia, Ley 1805 de 2016, modificó la legislación colombiana en cuanto a cómo se accede a un trasplante de órganos, pero, sobre todo, cambió la figura de donatario y dispuso el término derivado del derecho del consentimiento presuntivo. Este define la hipotética voluntad de una persona de ser donante de órganos como manifestación de solidaridad y beneficencia con otra persona en situación de necesidad y vulnerabilidad relacionada con su salud y las dimensiones que la definen. En el siguiente texto se presentan siete momentos que se consideran hechos fundamentales en la construcción de una cultura del valor de la atención en salud en la política nacional de trasplantes de Colombia.

## Abstract (en)

The national transplant law in Colombia, Law 1805 of 2016, modified the Colombian legislation regarding how a person accesses an organ transplant, but above all, it changed the donor figure, establishing the term derived from the presumptive consent right. This term implies a person’s hypothetical willingness to be an organ donor as a manifestation of solidarity and charity towards another person in a situation of need and vulnerability concerning his/her health and the dimensions that define it. In the following text, seven moments are considered fundamental facts when constructing a culture about the value of healthcare in the national transplant policy in Colombia.

La donación de órganos es un acto autónomo, gratuito, altruista e individual de la persona ^1^, regido por la manifestación de voluntad (el consentimiento legítimamente declarado) como artífice principal de la relación jurídica que nace cuando el donante vivo, de forma voluntaria, expresa su deseo de donar un órgano o algún otro componente anatómico de su cuerpo a otra persona ^2^.

La Ley Nacional de Trasplante de Órganos de Colombia, Ley 1805 de 2016, decreta en sus 22 artículos la política nacional de trasplantes, en especial, lo referente a la voluntad activa o presuntiva de la donación de órganos de cualquier ciudadano colombiano. Este hecho pretende generar un vínculo jurídico obligatorio y promover el aumento de las donaciones de órganos frente a la relación desproporcionada que existe entre los donantes activos o por presunción y la lista de pacientes que espera acceder a un trasplante ^1^.

La tesis central de este escrito propone que, en Colombia, se requiere la construcción colectiva y comunitaria -que incluya a todos los ciudadanos- de una cultura de educación sobre la política nacional de trasplantes en la que todos los actores sociales y políticos difundan y expandan el cuidado de la salud propia y de la del otro, sobre todo, lo relacionado con trasplantes de órganos y tejidos. Esta cultura debe ir más allá del cumplimiento de una norma o política: debe promover los valores de la compasión y la solidaridad. Sin embargo, estos hechos solo podrán construirse cuando la información disponible en la ley nacional se convierta en educación y cultura social promotora del valor de la vida, el cuidado de los otros y la solidaridad como camino ético para aumentar el número efectivo de trasplantes de órganos y tejidos en Colombia, y así, obtener mejores condiciones de salud y calidad de vida de la población.

Se presenta a continuación el desarrollo de los argumentos y contraargumentos de siete momentos considerados importantes para el desarrollo de la tesis propuesta.

## Voluntad de ser donante de órganos y tejidos

La posibilidad de una persona de ser donante se puede dar en dos situaciones. La primera es la del donante vivo de órganos o tejidos ante la necesidad de otra persona de recibir un órgano para garantizar su salud, su supervivencia o la mejoría en su calidad de vida. Esta forma de donación se denomina voluntaria, directa o consentida. La segunda es como donante fallecido, habiendo manifestado anticipadamente la voluntad de donar órganos ante el propio fallecimiento, como medida informada, voluntaria y reconocida por la familia del potencial donante.

Ambas situaciones están definidas por la voluntad de la persona, están contempladas por la legislación colombiana, y han sido adoptadas en los protocolos de atención clínica de los grupos de trasplantes de hospitales y clínicas de Colombia. Estas instituciones son prestadoras de servicios de salud en todos sus niveles de atención y realizan el componente técnico entre donante y donatario, según las normativas vigentes y la regulación establecida por el Instituto Nacional de Salud. Lo anterior, se hace para dar garantía al cumplimiento científico de la calidad y de la seguridad requeridas por un procedimiento especializado, como lo es un trasplante.

Sin embargo, la voluntad de ser donante vivo o fallecido implica temas éticos que se deben considerar durante la toma de decisiones de la persona, en uso del principio de autonomía, como la información previa a la donación entre las partes implicadas, la firma del consentimiento informado o el documento legal que legitima la voluntad expresada, las consecuencias médico-legales del procedimiento y la libertad de poder cambiar esta voluntad en cualquier momento del proceso de donación.

Para quien es donante vivo y va a ceder un órgano, tejido o componente vital, la información sobre la donación es un constructo que requiere tiempo de preparación para que él y su familia, con el acompañamiento del equipo de trasplantes, puedan asesorarse e informarse progresivamente sobre el procedimiento. Sucede de la misma manera con el donatario, quien en este caso se beneficia del procedimiento y también debe tener la información suficiente y completa, y el seguimiento continuo del equipo de trasplantes, para garantizar que potenciales donadores tomen decisiones informadas y tengan plena seguridad y confianza en el equipo médico tratante.

La información insuficiente para un donante vivo, así exista un vínculo familiar (genético) con el donatario, lleva a una situación de incertidumbre, desconfianza e inseguridad, incluso con el equipo de médicos tratantes, quienes pueden tener cierta premura por obtener la firma de un consentimiento informado. La persona en proceso de convertirse en donante puede carecer de suficiente claridad respecto al procedimiento, tener dudas, cuestionamientos iniciales y otras preguntas que surgen del proceso mismo. Estos interrogantes, que pueden ser repetitivos, y las respuestas solicitadas, deberían ser aclarados cuantas veces fuere o sea necesario. En este contexto, vale la pena hacerlo ya que el proceso concertado con el donante vivo es el que más tiempo toma antes de llevar a cabo el procedimiento. Por esta razón, se aspira a que la relación entre el donante y el equipo de médicos tratantes sea estrecha y personalizada, basada en la confianza y que permita retroceder o avanzar en la información cuantas veces sea necesario, para mejorar la comunicación y lograr un desenlace exitoso (para ambas partes) del procedimiento.

De acuerdo con lo promulgado en la Ley 1805 de 2016, la información para el donante fallecido debería ser tan anticipada como sea posible y, en esto, la divulgación de la Ley Nacional de Trasplantes juega un papel esencial, como se menciona en la misma:

“[...] El gobierno nacional, a través del Ministerio de Salud y Protección Social, o quien haga sus veces, implementará estrategias de información a la población que sean claras, objetivas, idóneas y oportunas sobre la existencia de la presunción legal de donación; las implicaciones de la ablación de órganos o tejidos; del derecho de oposición a la presunción legal de donación y los mecanismos para manifestarlo [...]”.

La forma como se realizan las estrategias educativas en Colombia sobre donación, trasplante e incluso voluntades anticipadas, son muy escasas ^3^, de poca difusión, de impacto social reducido y de pocos resultados en el objetivo de aumentar el número de trasplantes en Colombia. Según las estadísticas nacionales del Instituto Nacional de Salud, hasta septiembre de 2021, se habían obtenido 188 donantes de órganos y 977 donantes de tejidos; además, se habían realizado 660 trasplantes de órganos con donante vivo o cadavérico: 515 con donante cadavérico y 145 con donante vivo. En comparación con el año no pandémico del 2019, el promedio fue de 1.243 del total de trasplantes, lo que representó una reducción del 22 %, aproximadamente, en Colombia. Esto se debió a las dificultades por mantener los protocolos clínicos de trasplantes en la contingencia de la infección por SARS-CoV-2, que después fueron adaptados para mantener las actividades.

El compromiso del gobierno con la divulgación de la Ley Nacional de Trasplantes debería estar enfocado en su impacto sobre otros departamentos administrativos, como los de educación o comunicaciones, porque pareciera limitarse solo a un tema de salud y protección social, pero en realidad, es de interés general y debería conocerse desde la formación básica en la infancia, en el proceso de identificar la atención en salud desde nuestra cultura, como refiere Diego Gracia en su texto “La salud no es un puro proceso natural o fisiológico, sino un hecho cultural” ^4^. Este autor menciona que una de las principales referencias internacionales en el ámbito de la bioética, verdaderamente importante, es la educación ética de la sociedad: “Debería ser la asignatura principal en las escuelas”. En esta línea de pensamiento y citando a López ^5^:

“[...] El objetivo de conocer es llegar a saber, el objetivo de enseñar es hacer saber y actuar a otro. El objetivo de educar es conseguir que el educando se capacite para decidir y realizar sus proyectos y se convierta en agente actor y autor de estos [...]”.

Si educar capacita al individuo para tomar decisiones, hay que capacitarse para ser actor del “cuerpo” en todas sus dimensiones y, según Bourdieu ^6^, en su sentido más amplio, no definiéndolo simplemente como el mero organismo, el contorno del individuo o el espacio que este ocupa, sino también, como un producto social y, por tanto, influido por la cultura, las relaciones de poder, de dominación y de clase, siendo la educación una necesidad primordial de los seres humanos desde la infancia. Por esto, se propone que sea desde la educación previa a la escolarización (la que se da en casa y en el entorno infantil) y, luego, en la educación primaria (en la escuela o institucional), y continúe durante toda la vida del individuo, que se construyan valores culturales para el cuidado del cuerpo y la comprensión de la donación de órganos y tejidos.

La educación que lleva al individuo a ser agente en una potencial cultura del cuidado del cuerpo permite que el cuidado de sí mismo y del otro sea incorporado, llevado a la práctica y mantenido como un hábito. La información sobre trasplantes sin un contexto educativo, lanzada al público sin clasificación, podría ser contraproducente, ya que podría fomentar sentimientos de rechazo, indiferencia u omisión, e ir en contra del objetivo central del procedimiento de trasplante, por lo que es necesaria una fase preparatoria que solo se puede dar en clave de educación.

La cultura de educación para fomentar valores en las personas, no se contempla desde la mencionada Ley 1805 -en la que parece una función solo del Estado- y, aunque este es un buen comienzo, la verdadera y más sostenible meta es obtener la participación ciudadana y la coeducación (una construcción colectiva) de la atención en salud y la donación de órganos, para que la cultura de la donación de órganos sea un hecho posible, cultural e históricamente aceptado, y que se transmita a las generaciones venideras como un cuidado de la vida propia y de los otros, más allá de un acto solidario y altruista, pero casi obligatorio por la ley.

La difusión de la Ley Nacional de Trasplantes en Colombia debería tener el mismo alcance que las campañas publicitarias nacionales de conciencia ciudadana de gran impacto, como las de prevención de accidentes viales, minas antipersonas, secuestro y tráfico de personas, o incluso, temas recientes como la eutanasia y el aborto en Colombia que, aunque no tienen la difusión estatal deseada, se han vuelto tendencia en redes sociales y otros medios de comunicación por la polémica relacionada con los derechos ciudadanos y la vida en su inicio y su final. El tema del trasplante de órganos no tiene definitivamente este grado de exposición mediática y. aunque no es el medio de divulgación ideal ya que la información muchas veces está sesgada, incompleta o alejada de la realidad, se puede rescatar que lleva a que se toque el tema en los hogares y haya una reflexión personal y familiar.

En Latinoamérica, se ha presentado una notable evolución en torno a legislar dos de los instrumentos más importantes del ejercicio de la autonomía, como son el consentimiento informado y las voluntades anticipadas. La legislación de instrumentos no garantiza mayor autonomía en salud, aunque su existencia como reguladora de acciones para la toma de decisiones constituye el primer paso para su ejercicio ^7^. Siete países latinoamericanos cuentan con normativas para establecer voluntades anticipadas: Panamá (2003), Guyana Francesa (2005), México (2009), Argentina (2009), Uruguay (2009), Colombia (2014) y Costa Rica (2022). Estos países contemplan la mayoría de edad para expresar esta voluntad, a excepción de Colombia y Panamá, que también incluyen a menores de edad con capacidad para tomar decisiones. Sin embargo, según los resultados del trabajo de Álvarez *et al*., en el que participaron 533 profesionales de la salud en Colombia, se evidenció que este gremio posee poco conocimiento sobre el documento de voluntades anticipadas ^8^. Es indispensable capacitar a todos los profesionales de salud sobre el tema e implementar programas institucionales sobre la planificación de decisiones anticipadas.

Colombia puede considerarse el país latinoamericano más vanguardista en estos temas de voluntades anticipadas y consentimiento informado, pero es necesario adelantar esfuerzos intersectoriales para promover la educación o enseñanza multinivel y la difusión de las leyes y resoluciones disponibles en el país. Esto puede lograrse con estrategias pedagógicas que acerquen a la comunidad al trasplante de órganos, bajo la premisa de una divulgación que reconozca las necesidades del público por impactar, la importancia de una información adaptada y que responda a cerrar las brechas de comunicación existentes.

## Oposición manifiesta a ser donante de órganos

La oposición o negación de una persona a ser donante de órganos o tejidos puede darse en varias circunstancias clínicas, según la Ley 1805 de 2016:

“[...] Artículo 4°. Manifestación de oposición a la presunción legal de donación. Toda persona puede oponerse a la presunción legal de donación expresando su voluntad de no ser donante de órganos y tejidos, mediante un documento escrito que deberá autenticarse ante notario público y radicarse ante el INS. También podrá oponerse al momento de la afiliación a la Empresa Promotora de Salud (EPS), la cual estará obligada a informar al INS [...]”.

De esta manera, el desistimiento de una persona a la donación de órganos y tejidos debe quedar registrado como un documento legal ante notario. Sin embargo, este requisito legal es desconocido por la mayoría de las personas o familias que se ven enfrentadas a una situación de donación ante el fallecimiento de un familiar y quieren ejercer el derecho a la negación de donación, pero no pueden hacerlo o encuentran dificultades por desconocer el documento o la voluntad previa del familiar o paciente, teniendo que enfrentar momentos de frustración, angustia y temor; por esto, en estos casos no hay una información previa suficiente que impida la toma de decisiones de forma apresurada y con incertidumbre en el momento emergente.

En estos casos es muy importante el conocimiento de la resolución nacional de voluntades anticipadas ^3^ que facilita la expresión del testamento vital de cualquier ciudadano:

“[...] Es el instrumento que le permite a la persona participar en la toma de decisiones relacionadas consigo mismo y el cuidado o atención de su salud, en el caso de que se encuentre incapacitado o limitado para proyectar su posición, o manifestar sus preferencias al final de la vida como consecuencia de un evento de salud que impide la expresión de la voluntad [...]”.

El testamento vital es un documento que puede suscribir cualquier persona capaz, sana o en estado de enfermedad, en pleno uso de sus facultades legales y mentales, y con total conocimiento de las implicaciones de esa declaración. Este documento se puede hacer y modificar las veces que la persona considere necesario, realizando adiciones o sustituciones al escrito de voluntad anticipada, y debe presentarse al médico tratante, ante dos testigos firmantes o ante un notario. El artículo 4 “Contenido del documento de voluntad anticipada”, del parágrafo 1 de la resolución, menciona la voluntad anticipada de donación y recomienda que sea un tema por tratar, incluso con personas sin enfermedad declarada, ya que facilita las intervenciones médicas del equipo de trasplantes en caso de que sea necesario iniciar un protocolo de donación urgente.

En este apartado toma vital importancia el concepto ético del respeto por la autonomía de la persona, el fundamento filosófico de la autonomía, tal como lo interpretaron los filósofos Immanuel Kant (1724-1804) y John Stuart Mill (1806-1873), y es que todas las personas tienen un valor intrínseco e incondicional y, por lo tanto, deben tener el poder para tomar decisiones racionales y elecciones morales ^9^.

En medicina, el respetar el principio de autonomía obliga al médico a revelar la información médica y las opciones de tratamiento que sean necesarias para que el paciente ejerza la autodeterminación, y apoye el consentimiento informado, la verdad y la confidencialidad. No obstante, esto exige tener acceso a una información completa, adaptada a un proceso educativo, para garantizar que el paciente como ser autónomo, tome una decisión con pleno conocimiento, en uso de sus facultades y capacidades, lo que le permite retroalimentar y construir y reconstruir la información si es necesario.

En el caso de donantes vivos, el proceso de retiro voluntario de un donante es una renuncia que se basa en el respeto de la autonomía del potencial donante de órganos. Esta voluntad debe partir de una información adecuada para la acción, que no es más que exponer plenamente a la persona autónoma, los efectos y consecuencias de su decisión. Deben conocerse los motivos de la negativa del donante de forma libre y sin coerción, para aclarar cualquier sombra en la decisión que limite el curso de la donación debido a información errónea, no verificada, creencias o mitos. La oposición manifiesta es un acto en el que es importante resaltar la educación en la ley de donación, porque es tan válido promover que haya donaciones como también evitar que haya detractores de estas por una debilidad en la estructura educativa requerida para la toma de decisiones.

## Abstención frente al tema o por desconocimiento de la ley

Otra posibilidad que puede presentarse frente al tema de trasplantes en Colombia es que las personas, aun conociendo la ley, decidan no expresar su voluntad frente a este tema, no tramiten su desistimiento ante un notario, no manifiesten su voluntad anticipada o que la ley sea mal interpretada o aplicada parcialmente. Todas estas situaciones llevan a que en la práctica clínica haya muchas dificultades para llevar a cabo el procedimiento en un servicio de urgencias o en un ambiente clínico emergente sujeto a mucha carga emocional entre familiares y el equipo médico tratante. En todos estos casos, es necesario conocer la normativa vigente y sostener conversaciones difíciles sobre temas al final de vida. Esto puede ser impulsado desde la atención primaria en salud como medida preventiva y, en esto, la Ley Nacional de Cuidados Paliativos, Ley 1733 de 2014 ^10^ se queda corta en proponer acciones en salud desde la atención primaria para difundir temas relacionados con el final de la vida y las voluntades previas relacionadas con este tema. Este es un foco para crecer como sociedad y formar comunidades de cuidado y solidaridad desde la atención básica en salud; algunas propuestas en esta línea tienen que ver con el desarrollo de ciudades compasivas o comunidades saludables ^11^:

“[...] Desde los años 90 se propuso un modelo teórico de ciudades o comunidades saludables que se basa en un proceso de participación ciudadana para lograr ese fin. La Organización Mundial de la Salud adoptó dicho modelo como una forma de aplicación de la promoción de la salud a la salud pública, en la cual busca que las autoridades gubernamentales, las instituciones de salud y bienestar, las organizaciones públicas y privadas y la comunidad en general, hagan esfuerzos conjuntos para mejorar las condiciones de vida, las relaciones armoniosas con el ambiente e incrementar los recursos de la comunidad buscando una mejor convivencia a través de la solidaridad y la cohesión [...]”.

En ese sentido, la creación de redes de cooperación constituye un eje central del modelo del desarrollo de las llamadas “comunidades compasivas” ^12^. Este movimiento ha dado lugar a que se pongan en marcha diferentes iniciativas en el mundo para promover e integrar socialmente los cuidados paliativos en la vida cotidiana de las personas, creando comunidades que cuidan y enseñan a cuidar a las personas al final de la vida. Esta iniciativa integra los esfuerzos de entidades públicas y privadas y de organizaciones no gubernamentales, para generar redes de ayuda y cooperación entre comunidades para la atención de las personas vulnerables o con enfermedades crónicas.

En Colombia, hay experiencias de estas iniciativas en siete ciudades compasivas que están enfocadas en el trabajo colaborativo sin ánimo de lucro en universidades, hospitales y centros comunales enfocados en promover una cultura de solidaridad ^13^. En estas comunidades en crecimiento podría enfocarse el tema de trasplante de órganos, pero requieren mayor financiación gubernamental y privada para convertirse en un verdadero movimiento social que cambie el paradigma del cuidado.

## Consentimiento informado explícito

La donación y el trasplante de órganos hacen parte del procedimiento médico que consiste en la sustitución de un órgano humano por otro proveniente de un donante humano. Este procedimiento requiere del cumplimiento de determinadas exigencias normativas, entre las cuales está el consentimiento voluntario del donante mediante el cual manifiesta su decisión voluntaria de contribuir en el procedimiento.

Algunos aspectos importantes relacionados con el procedimiento tienen que ver con seguridad, información, competencia, motivación solidaria, ausencia de coacción y lucro, y consentimiento libre, voluntario y expreso. Es importante mencionar que la ausencia de coacción es indispensable para que la decisión sea propiamente fruto de la autodeterminación de la persona y que manifieste la ausencia de factores coactivos externos (Ferriol ALC, Mora LM, Fernández DMH. El consentimiento informado en el trasplante de riñón de donante vivo: una aproximación a aspectos médicos-legales. Sid Cu. Segundo Congreso Virtual de Ciencias Básicas Biomédicas en Granma. Manzanillo, México, 2021. Disponible en: https://cibamanz2021.sld.cu/index.php/cibamanz/cibamanz2021/paper/viewFile/124/113.

El consentimiento informado explícito aplica principalmente para personas que han tomado la decisión de realizar voluntariamente una donación de órganos para beneficiar a un familiar, pariente o conocido, que está en la lista de trasplantes y que cumple con las características o el perfil previamente determinados por el grupo de trasplantes. La firma de este documento legal requiere que el equipo tratante garantice una comunicación lo más cercana posible en todos los aspectos relacionados con el trasplante y las consecuencias del procedimiento, que estas estén totalmente claras y se pueda desarrollar una relación de confianza entre equipo médico, donador y su familia, y donatario y su familia.

Según la Ley 1805 de 2016, es claro que quien no se opone a la donación de órganos se supone un donador voluntario o que se asume su consentimiento presuntivo. Para ser este tipo de donador no es necesario registrar en una notaría el deseo personal de hacerlo, basta con hacer un registro en la página del Instituto Nacional de Salud (https://apps.ins.gov.co/CarneDonantes/Aspx/Registro/frmDonante.aspx) para obtener el carné de donante; también, se puede actualizar la información e, incluso, retirar esta voluntad de donación. La página web está disponible para cualquier persona que tenga acceso a internet en cualquier parte del mundo (aplica para ciudadanos colombianos), sin embargo, esto limita el acceso porque no garantiza una cobertura de personas sin acceso a internet o sin conocimientos de este. Esta situación invita a pensar en la posibilidad de extender la política de trasplante de órganos a la atención primaria, pero no solo como una campaña aislada, sino como una estrategia de atención permanente, continua y sostenida que facilite un “clima social y comunitario de donación”.

## Consentimiento informado presuntivo

Con la Ley 1805 de 2016 del Congreso de Colombia, se inició una transformación en la legislación colombiana frente a la donación de órganos, tejidos, componentes anatómicos y líquidos orgánicos. Esta ley pasó a modificar la Ley 73 de 1988 y la 919 del 2004, y busca principalmente ampliar el concepto de presunción legal de donación ya que, en las versiones legislativas previas a la ley del 2016, ya se contaba con el concepto de “consentimiento del donante, del receptor, consentimiento de los deudos o abandono del cadáver”. La Ley 1805 adopta el término consentimiento presuntivo, traído del derecho penal ^14^, citado como

“[...] un juicio de probabilidad objetivamente judicial en que el afectado, si tuviese pleno conocimiento del estado de cosas y desde su punto de vista personal, hubiese consentido la conducta [...]”.

El consentimiento se genera en los supuestos en los que el titular del bien jurídico se ve imposibilitado de consentir (manifestar su voluntad), presumiéndose su consentimiento en la hipótesis o ficción de que él habría consentido en caso de conocer el hecho y tener la ocasión para hacerlo ^15^.

El problema es que en la práctica realmente no existe un consentimiento, sino una suposición de que el titular del bien jurídico, de conocer las circunstancias específicas, lo hubiese prestado, razón por la que no puede afirmarse que en estos supuestos el individuo haya hecho uso de su autonomía de la voluntad para tomar una decisión ^15^.

Aunque la figura en cuestión es reconocida por la jurisprudencia como una causal autónoma de justificación, su sentido o carácter de independiente es controvertido ^14^.

En el consentimiento presuntivo se interfiere sin permiso y, por ello, realizando el tipo delictivo en los bienes jurídicos de otro, el consentimiento presunto es una construcción normativa, mientras que el consentimiento efectivo, activo o explícito es una manifestación de voluntad en línea similar ^16^. Frister hace uso del término “voluntad hipotética” para referirse a un consentimiento de la persona que no se expresa directamente, luego no puede considerarse un hecho legítimo de su autonomía ^17^.

La Corte Constitucional de Colombia, mediante la sentencia C-933 de 2007, precisó que la presunción legal para la donación de órganos se encuentra fundamentada en los principios de libertad y solidaridad social y, del mismo modo, aclaró que esta figura debe respetar el derecho de los familiares a oponerse al procedimiento de extracción de órganos del cuerpo del familiar fallecido ^18^.

En 2017, Kcomt expresó que es preferible utilizar el término voluntad presunta en lugar de consentimiento presuntivo, a fin de evitar confusiones terminológicas, sobre todo cuando la voluntad presunta, a diferencia del consentimiento, no operaría como una causa de atipicidad, sino como una causa de justificación autónoma ^15^.

El modelo de consentimiento presunto está lejos de estar éticamente exento de problemas y más bien está plagado de críticas, en particular, sobre su eficacia para superar la escasez de órganos ^19^, porque se convierte en una medida forzada o impuesta que obliga a los ciudadanos y no promueve una solidaridad o compasión que se origine de un proceso educativo, de una cultura del cuidado.

“[...] El consentimiento presunto es sin duda un instrumento jurídico que contribuye de forma significativa en el desarrollo de programas médicos de trasplante de órganos y componentes humanos de manera benéfica y efectiva para los pacientes en espera, con necesidades terapéuticas o de tratamiento” [...] ^18^.

Sin embargo, no se puede predicar la eficacia de la norma que hace extensiva la voluntad del donante, cuando este no ha sido previamente informado de este derecho,

“[...] el régimen normativo de la presunción legal del donante solo podrá ser efectivo en la medida en que exista suficiente pedagogía sobre su validez [...]” ^18^.

Visto de esta manera, el consentimiento presuntivo como medida jurídica lleva inherente algunos problemas éticos y jurídicos que, en muchas ocasiones, son el reflejo de deficiencias del Estado en su incapacidad institucional de transformar el pleno conocimiento de la norma en una herramienta pedagógica. Por ello, se requiere la cooperación interinstitucional, entre organizaciones públicas y privadas -no solo en el ámbito de la salud- para que estratégicamente unidas faciliten la expansión de la política pública de trasplante a nivel nacional.

## Listas de espera de pacientes frente a los recursos limitados

El tiempo que una persona permanece en lista de espera para recibir el órgano es un periodo de inseguridad por la posibilidad de ocurrencia de acontecimientos impredecibles porque la demanda de trasplantes es mayor que la disponibilidad de donantes ^20^. El tiempo de espera para llegar a un trasplante suele ser indeterminado y, en el proceso, los pacientes suelen aumentar su carga sintomática y de la enfermedad, están sometidos a una gran presión psicológica, hospitalizaciones, trámites administrativos, negaciones de autorizaciones médicas y tensión acumulada, lo cual genera un cambio en las dinámicas familiares y una alteración de la calidad de vida del paciente y su familia acompañante.

Los criterios para la asignación de órganos a los pacientes en lista de espera deben ser totalmente objetivos y exclusivamente médicos, buscar el máximo beneficio del receptor ^21^, de carácter público por si fuere necesario dar cuenta del proceso de la asignación, ser aceptados con el mayor consenso posible y basarse en normas de actuación que tengan entre sus objetivos el elegir.

Existe una obligatoriedad de deliberación en los comités de ética hospitalarios o institucionales antes de la práctica de un trasplante renal con donante vivo y en el caso de trasplante de médula ósea alogénico ^22^. En el análisis ético de los casos, media el gesto altruista del donante vivo y sano de riñón, amparado en el principio de autodeterminación y voluntad de una decisión personal, sin presiones y basada en la generosidad, la autonomía, la libertad y la gratuidad ^23^.

La deliberación en el proceso de donación tiene en cuenta criterios éticos y dimensiones vitales, como la prioridad o urgencia que puede existir entre un paciente y otro, el beneficio terapéutico potencial, las características médicas del receptor y la antigüedad en la lista de espera. La urgencia habría que entenderla como la condición clínica de un paciente que requiere del tratamiento con trasplante de órgano en un periodo que evite la muerte ante la ausencia de otras alternativas terapéuticas.

El escoger de una lista de espera de manera aleatoria a los pacientes y sin la aplicación de ningún criterio ético o médico sería inaceptable, dado que se requieren el discernimiento y el análisis de las condiciones individuales de cada caso particular. Aunque se debe respetar la lista de espera, no todos los órganos disponibles tienen las características para sus donatarios o receptores y se requiere equilibrar -en un ejercicio deliberativo- aspectos como la antigüedad en la lista de espera frente a la compatibilidad y las condiciones del receptor.

Es inaceptable aplicar algunos criterios, como aquellos mercantilistas, la inclusión de un paciente en más de una lista de trasplantes, la exclusión de pacientes por estigma social o por prácticas cuestionadas en perjuicio de su propia salud. Cada caso es una necesidad y merece toda la atención sanitaria y social; no se debe aceptar la exclusión de ciertas entidades y pacientes, de los tratamientos con trasplantes. En su artículo, De Frutos se refiere a la donación de órganos como “una alianza rentable”, “no obstante, es preciso concretar las exclusiones a la aceptación para trasplante, siempre basadas en razonamientos objetivos y científicamente probados” ^21^.

## Justicia distributiva o la equidad en la distribución

Partiendo de la relación desproporcionada entre el número de órganos para trasplante y la reducida lista de espera, resulta indiscutible que se debe hacer una distribución entre los pacientes receptores, según sus características de enfermedad de base, edad, condiciones de compatibilidad, expectativa de vida calculada y factores que pueden modificar esta supervivencia.

La edad como un criterio en la escogencia de un donador y un donatario, es un tema ético con muchos matices. En el presente, se prefiere una primera asignación de donantes jóvenes para receptores jóvenes y donantes mayores para receptores mayores, lo que se denomina *age matching u old for old*^24^.

El criterio de la antigüedad en la lista de espera del trasplante, entendido como el orden de llegada, es tenido en cuenta y valorado en la distribución de cualquier bien que se considere escaso ^21^, es una categoría fundamental de acceso a un servicio o producto. Cada lista de espera de trasplante tendrá que establecer su abanico de antigüedad, bien sea mediante una distribución por cuartiles u otra estratificación, o por medio de alguna ponderación que equilibre a los más antiguos respecto a los recién llegados ^21^.

Todos los criterios aplicados en la asignación de un trasplante deberían apuntar hacia el aprovechamiento de los recursos disponibles y la justicia distributiva. De acuerdo con los principios generales de bioética del Informe Belmont, el criterio de justicia es fundamental ^25^ y consiste en dar a cada cual, según su derecho o imparcialidad, la distribución de un recurso o un tratamiento; es la igualdad de oportunidades y la responsabilidad ante terceras partes que exigen máxima moralidad en la asignación justa de unos recursos limitados (órganos) que la sociedad, en general, le ha confiado para cumplir un fin ^21^.

Ortúzar menciona que

“[...] el caso del trasplante de órganos sería un ejemplo de escasez natural débil, es decir, se considera que no hay nada que alguien puede hacer para aumentar la oferta de órganos al punto de satisfacer a todos.

En segundo lugar, un bien indivisible porque es imposible que lo reciba más de una persona, característica propia de los órganos para trasplantes.

En tercer lugar, un bien heterogéneo porque no todos los órganos serán de igual calidad, existirán algunos mejores que otros ^26^.”

Algunos autores, como Tom Koch, consideran que el trasplante de órganos es un caso de escasez absoluta, aquel en el cual la demanda supera absolutamente a la oferta (algunos pacientes en lista de espera morirán sin importar cuál sea la política seguida) ^27^.

Para intentar solucionar muchas desigualdades en el tema de trasplantes, es necesario redistribuir los bienes en la forma más equitativa posible y con enfoque en el individuo, para no reproducir las injusticias generadas ^26^. La concepción de Rawls de justicia como equidad se halla en su preocupación por defender los derechos del individuo y la redistribución desigual e imparcialidad de los bienes a favor de los más desfavorecidos ^28^. Sin embargo, si se aplica en forma estricta el principio de este autor, el único criterio sería brindar oportunidades solo al peor situado y esto no contribuiría a la equidad.

Desde una concepción de justicia como equidad, es necesario compensar las injusticias producto de múltiples factores para evitar las desigualdades sociales ^26^. Por lo tanto, no sería justo considerar exclusivamente la eficiencia como valor para distribuir los órganos, sin considerar la equidad y el significado social de la atención médica: atender a los más necesitados y no a los más sanos. La clave está en lograr el equilibrio entre la equidad y la eficiencia, es decir, llegar a una distribución más centrada en las necesidades de cada persona, individualizando cada situación en un marco de salud colectiva, con un respeto personal y un cuidado de la comunidad en un fino equilibrio entre necesidades y recursos disponibles.

## Conclusión

La ley nacional en Colombia sobre trasplante de órganos es una oportunidad de ejercicio ciudadano de derechos para el receptor y el donante de órganos, que permite la ejecución de un procedimiento médico, voluntario o de voluntad sustituta, autónomo y altruista, con base en la solidaridad. Desde la ley, se propone la divulgación nacional de interés para todos los colombianos, dada la falta de mecanismos de difusión y comunicación con interés educativo y transversal a toda la comunidad.

El cambio reciente hacia la implementación de un consentimiento presunto con la Ley Nacional de Trasplantes del año 2016 en Colombia implica que desde la norma haya una imposición y una obligación para donantes y donatarios. Es una relación que no está basada en un proceso de educación y cultura ciudadana hacia la construcción de una cultura sobre el valor de la atención en salud y, por lo tanto, genera muchas dificultades, como falta de confianza y certeza en la relación que se establece entre el equipo médico, el donante y su receptor potencial, y las familias implicadas.

A partir de este escrito, se sugiere propender desde la política pública y la integración intersectorial, a un sistema más solidario para las familias y los equipos de trasplante, sin intereses contrapuestos, que vaya más allá de perseguir un enfoque de consentimiento presunto bajo un mandato legal, y que permita el desarrollo de una conciencia social y ciudadana de cuidado desde la educación y la construcción de valores personales y familiares para una mejor calidad de vida y salud de la comunidad.
